# Concentric Ring Probe for Bioimpedance Spectroscopic Measurements: Design and Ex Vivo Feasibility Testing on Pork Oral Tissues

**DOI:** 10.3390/s18103378

**Published:** 2018-10-10

**Authors:** Shekh Emran, Reijo Lappalainen, Arja M. Kullaa, Sami Myllymaa

**Affiliations:** 1 SIB Labs, Department of Applied Physics, University of Eastern Finland, P.O. Box 1627, FI-70211 Kuopio, Finland; shekh.emran@uef.fi (S.E.); reijo.lappalainen@uef.fi (R.L.); 2 Institute of Dentistry, University of Eastern Finland, P.O. Box 1627, FI-70211 Kuopio, Finland; arja.kullaa@uef.fi; 3 Research Unit of Oral Health Sciences, University of Oulu, P.O. Box 8000, FI-90014 Oulu, Finland; 4 Educational Dental Clinic, Kuopio University Hospital, P.O. Box 100, FI-70029 Kuopio, Finland

**Keywords:** bioimpedance spectroscopy, electrical impedance spectroscopy, electrical characterization, probe, soft tissue, oral cancer, potentially malignant disorders, oral mucosa

## Abstract

Many oral diseases, such as oral leukoplakia and erythroplakia, which have a high potential for malignant transformations, cause abnormal structural changes in the oral mucosa. These changes are clinically assessed by visual inspection and palpation despite their poor accuracy and subjective nature. We hypothesized that non-invasive bioimpedance spectroscopy (BIS) might be a viable option to improve the diagnostics of potentially malignant lesions. In this study, we aimed to design and optimize the measurement setup and to conduct feasibility testing on pork oral tissues. The contact pressure between a custom-made concentric ring probe and tissue was experimentally optimized. The effects of loading time and inter-electrode spacing on BIS spectra were also clarified. Tissue differentiation testing was performed for ex vivo pork oral tissues including palatinum, buccal mucosa, fat, and muscle tissue samples. We observed that the most reproducible results were obtained by using a loading weight of 200 g and a fixed time period under press, which was necessary to allow meaningful quantitative comparison. All studied tissues showed their own unique spectra, accompanied by significant differences in both impedance magnitude and phase (*p* ≤ 0.014, Kruskal-Wallis test). BIS shows promise, and further studies are warranted to clarify its potential to detect specific pathological tissue alterations.

## 1. Introduction

Many potentially malignant disorders of the oral cavity, such as oral leukoplakia, erythroplakia, and oral lichen planus, cause abnormal structural changes in the oral mucosa [1]. In clinical practice, diagnostic tests available for these changes include visual inspection, palpation, staining with toluidine blue, oral brush biopsy, and scalpel biopsy coupled with histological examination. On the other hand, the diagnosis of oral mucosal lesions (both malignant and benign) currently relies on surgically removed biopsies. This invasive procedure causes pain and discomfort for the patient. The procedure is often stressful for both the patient and the operator [2]. Furthermore, the histopathological assessment of biopsies is time-consuming and expensive [3]. Thus, there is an increasing demand for the development of methods for diagnosing various oral diseases by means of non-invasive and painless oral mucosal measurements.

Electrical impedance spectroscopy (EIS) is a powerful technique for assessing the electrical characteristics of material as a function of the frequency of an applied electrical current. Electrical impedance is a delicate marker of minor changes in natural materials and particularly in biological tissues, such as mucous membranes, skin, and integuments of organs [4]. Therefore, several researchers worldwide have tried to find convenient EIS-based solutions to detect and quantify pathological tissue alterations [5]. For example, bioimpedance spectroscopy (BIS) has been utilized for the evaluation of skin sores [6] and the assessment of muscle health in patients with neuromuscular disorders [7]. Techniques based on electrical impedance tomography can aid the assessment of ischemic coronary illness and aspiratory edema [8,9]. Recently, these impedance-based approaches have been progressively utilized as a part of the discovery of tumors in various tissues such as skin, breast, and female reproductive organs [10]. Recently, Tatullo et al. [11] designed a four-terminal intraoral probe for the characterization of healthy and clinically oral lichen planus affected oral mucosa. They concluded that bioimpedance could be a valid aid in the early detection and clinical monitoring of the suspicious lesions.

The utilization of BIS has recently been built up in dentistry using diverse pinnacle locators for the root trench length assurance [12]. However, the potential of employing BIS for the diagnostics of oral mucosal diseases has not been studied in depth [13]. Biological tissues (cells, intra- and extra-cellular space, matrices) contain components having both resistive and capacitive properties, resulting in a complex electrical impedance when a low-intensity electric current is applied to the tissue [14]. Both the magnitude of the impedance and other electrical parameters and their dependence on frequency are related to tissue composition, and thus different tissue structures are associated with different frequency bands within an impedance spectrum [15]. A BIS analysis conducted over a wide frequency range and utilizing various measurement depths could provide detailed information about the tissue interiors, which help us to understand better the anatomy, physiology, and pathology of biological tissues. This is crucial in developing novel non-invasive tools for tissue characterization, diagnosis of various disorders and monitoring degenerative changes related to different diseases and follow up of post-treatment outcomes. Overall, there exists abundant research related to skin measurements with BIS [10,16,17,18,19]. However, the measurements of oral tissues are much rarer [20].

The aims of this study were (i) to design a new concentric ring probe for BIS measurements, (ii) to optimize its function and reproducibility for soft tissue measurements, and (iii) to test its suitability for tissue discrimination in extracted pork oral tissue samples. We hypothesized that the applied probe contact pressure on tissue must be optimized and kept constant to enable repeatable measurements. We also hypothesized that the loading time has a clear effect on the obtained data, and this parameter needs to be fixed as well. Finally, we hypothesized that the optimized measurement setup is capable of distinguishing different types of ex vivo pork oral tissue samples.

## 2. Materials and Methods

### 2.1. Design of the Concentric Ring Probe

**Figure 1:**
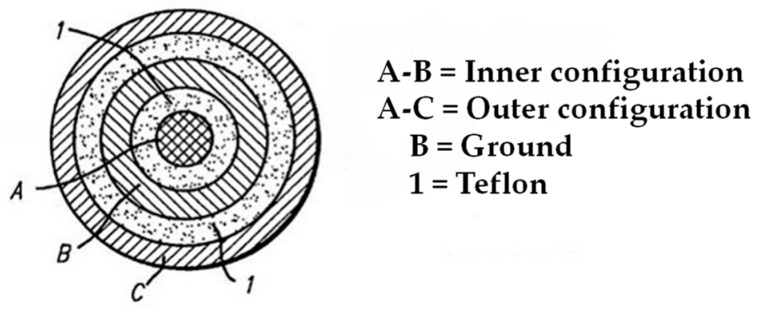
A plane top view of the tip of a probe with two measuring ring electrodes around the central pin electrode. In the inner configuration, the voltage is applied between electrodes A and B, whereas in the outer configuration the voltage is applied between electrodes A and C (with B is acting as a ground).

A concentric ring probe was designed based on the previously introduced principles by Ollmar (1991) [5] and Richter et al. (2015) [13]. It was originally designed for oral tissue biopsy measurements (the intraoral mucosal sample is 8 mm in its diameter). It is composed of two ring-shaped, stainless-steel electrodes (inner/outer diameter: 3.5 mm/5.0 mm and 6.5 mm/9.0 mm) around a central pin with a diameter of about 2 mm (Figure 1). Teflon was used as an insulator material.

This probe, about 9 mm in diameter and about 30 mm in length, can be placed on ex vivo tissue samples in a simple mechanical setup to ensure a proper pressure on a biopsy with a diameter of 8 mm. Figure 1 shows a plane top view of the tip of the probe, in which letter ‘A’ indicates the center pin electrode, ‘B’ indicates the inner ring electrode, and ‘C’ indicates the outer ring electrode. In the inner configuration, the voltage is applied between electrodes A and B, whereas in the outer configuration the voltage is applied between electrodes A and C. The outer configuration with grounding is similar to the regular outer configuration, except that electrode B is connected to a ground terminal to eliminate leakage (surface) current.

### 2.2. Measurement Setup

**Figure 2:**
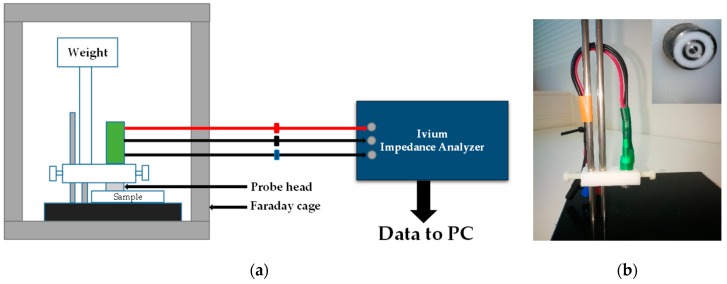
(**a**) Schematic overview of the experimental setup; (**b**) custom-made concentric ring probe with the surface of the probe head shown in the upper right corner of the picture.

A custom-made concentric ring probe with a commutative loading weight (100 g, 200 g, or 400 g) was placed in an aluminum box (i.e., a Faraday cage) in order to decrease external electromagnetic interference (noise) (Figure 2). The measurement cables were passed through the Faraday cage and connected to a CompactStat.h: Portable Electrochemical Interface and Impedance Analyzer (Ivium Technologies, Eindhoven, The Netherlands).

The BIS data were collected and stored using a laptop running IviumSoft Electrochemistry Software (Ivium Technologies). The frequency range for the sinusoidal excitation signal was set between 1 Hz and 3 MHz. The AC voltage was kept as a constant (50 mV), whereas the current varied based on impedance (ranging up to 10 mA) during the measurement procedure.

### 2.3. Optimization of the Measurement Protocol

**Figure 3:**
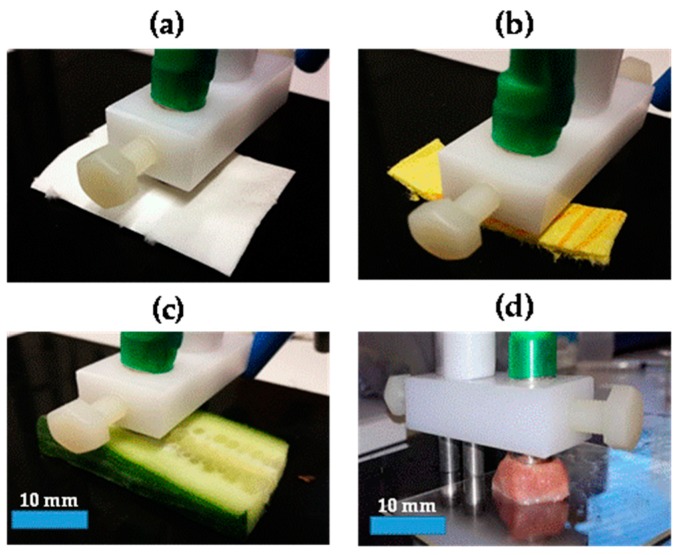
Non-biological samples, i.e., white tissue paper (**a**) and yellow towel (**b**), and biological samples, i.e., cucumber (**c**) and pork tongue (**d**), under testing.

Various synthetic and biological materials were used as phantom materials for testing and optimizing the concentric ring probe and for the overall measurement protocol. In the first part of the study, we used two non-biological samples (i.e., white tissue paper and yellow towel (Figure 3)) as phantom materials. We measured the BIS data without using a loading weight, as well as with a loading weight of 100 g, 200 g, and 400 g. Furthermore, we used three optional measurement configurations: inner, outer, and outer with grounding (see Figure 1). Before the measurements, we moistened the samples with a few drops of physiological saline solution (Natrosteril 9 mg/mL). Relative standard deviations (RSDs; also termed coefficient of variation, CV) for repeated BIS measurements (*n* = 3) with various loading weights and configurations were calculated.

After that, the BIS data were measured for two biological samples, i.e., cucumber and pork tongue (Figure 3). The BIS data were measured considering four areas for each sample using inner and outer with grounding configurations with a fixed loading weight (200 g). Five BIS scans were conducted, and each measurement took approximately 2 min, with a 1-min break before the next measurement. Complex divisions of repeated spectra were determined to make intra-sample variability and loading time effect issues easier to interpret. The complex divisions were calculated by dividing the latter impedance magnitude spectra by the first one and subtracting the latter phase spectra by the first one.

### 2.4. Tissue Differentiation with Ex Vivo Pork Oral Samples

To clarify the capability to distinguish different tissue types, we measured BIS spectra for ex vivo pork oral tissue samples. Porcine jaw samples, extracted from two animals, were taken from the freezer and immediately after thawing, different types of tissue including palatinum, buccal mucosa, fat and muscle samples were excised (Figure S1). Tissue samples were stored in a box with towels wetted with physiological saline solution until measurement. All measurements were performed on the same day over a few hours to reduce dehydration changes. BIS spectra were measured considering up to six locations for each sample. Both inner and outer with grounding configurations together with a fixed loading weight (200 g) were used in all tissue measurements.

### 2.5. Statistical Analysis

The magnitude of impedance (|*Z*|), parallel resistance $\left(R_{p}\right)$, parallel capacitance ($C_{P})$, and phase angle (*θ*) were measured between 1 Hz and 3 MHz, starting from the highest frequency. From these measurements, relative permittivity ($\epsilon_{r}^{\prime }$), loss factor ($\epsilon_{r}^{\prime \prime }$), dissipation factor (tanδ), and conductivity ($\sigma^{\prime }$) were determined by using Equations (1)–(4).

(1)Relative permittivity          ϵr′=CPCe(2)Loss factor          ϵr″= 1RPjωCe(3)Dissipation factor          tanδ= ϵ″ϵ′(4)Conductivity          σ′= ϵ0RpCe

Explanation of the parameters used in Equations (1)–(4):



$C_{P}$

Parallel capacitance

$C_{e}$

Capacitance of an empty measuring cell

$R_{p}$

Parallel resistance

ω

Angular frequency

$\epsilon_{0}$

Permittivity of free space = 8.854 × 10^−12^ F⋅m^−1^

A non-parametric statistical test, the Kruskal-Wallis test, followed by Dunn’s post hoc analysis, was used to investigate the significance of differences in the BIS parameters (impedance magnitude and phase) between the different tissue types. A *p*-value ≤ 0.05 was considered statistically significant. Statistical analyses were conducted either with Microsoft Excel or with SPSS software (version 23.0; SPSS, Chicago, IL, USA).

## 3. Results

### 3.1. Optimization of the Measurement Protocol with Different Phantom Materials

The effect of loading weight and electrode configuration (inter-electrode spacing) on BIS spectra and reproducibility was tested using white tissue paper and yellow towel as phantom materials. Spectra were highly similar in their shapes, except for that of outer with grounding configuration, without any additional weight that resulted in highly anomalous spectra. In all cases, there was a rapid drop in impedance magnitude and an increase in phase at the frequency around 1 MHz. The RSDs for repeated BIS measurements with various loading weights are shown in Figures S2 and S3. Generally, using a heavier loading weight, i.e., either 200 g or 400 g, resulted in lower RSD values compared to the lighter weight (100 g or no weight). Furthermore, the inner configuration and outer configuration with grounding resulted in lower RSD values, thus having better reproducibility compared to the outer configuration.

### 3.2. Effect of Loading Time

**Figure 4:**
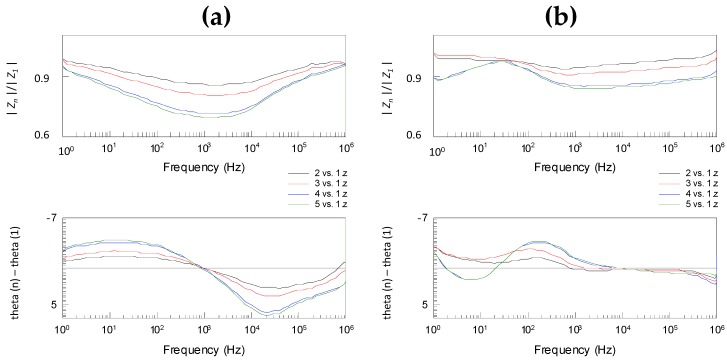
The complex division of bioimpedance spectroscopy (BIS) spectra for the same (**a**) cucumber and (**b**) tongue sample using the inner configuration shows the clear time-variant nature of the measurements. A total of five repeated scans were performed, and here the subsequent measurement is compared to the first one.

**Figure 5:**
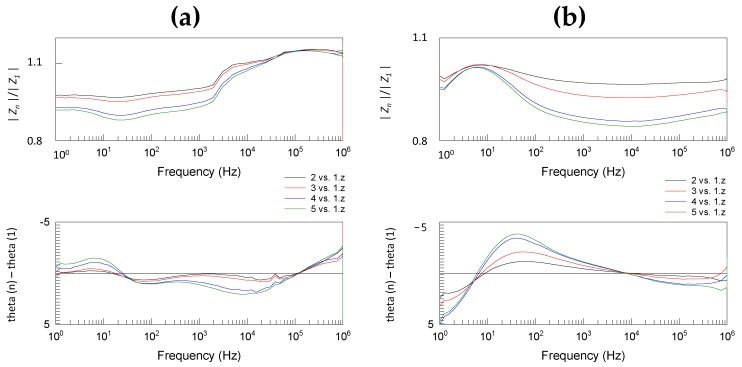
The complex division of BIS spectra for the same (**a**) cucumber and (**b**) tongue sample using the outer with grounding configuration shows the clear time-variant nature of the measurements. A total of five repeated scans were performed, and here the subsequent measurement is compared to the first one.

We observed that the loading time has a clear effect on obtained impedance spectra (Figure 4 and Figure 5, corresponding raw data is shown in Figures S4 and S5). The complex division of repeated BIS spectra for the same sample of cucumber or pork tongue showed a clear time-variant nature of the measurements. Using the inner measurement configuration (Figure 4), the effect of loading time was more intense in the cucumber sample measurements than that in the tongue sample measurements.

In the case of cucumber, the fifth measurement produced over 30% lower impedance magnitude at 1 kHz compared to the first measurement, whereas the corresponding difference was only ~15% in the case of tongue. The loading time effect in the tongue sample measurements was found to be substantial and equal between the inner (Figure 4) and outer with grounding configurations (Figure 5). Instead, the effect in the cucumber sample measurements was remarkably weaker when the outer with grounding configuration was used. In this case, the fifth measurement produced 12% lower impedance magnitude and this maximum weakening was occurred at lower frequency, i.e., at 25 Hz.

### 3.3. Tissue Differentiation

**Figure 6:**
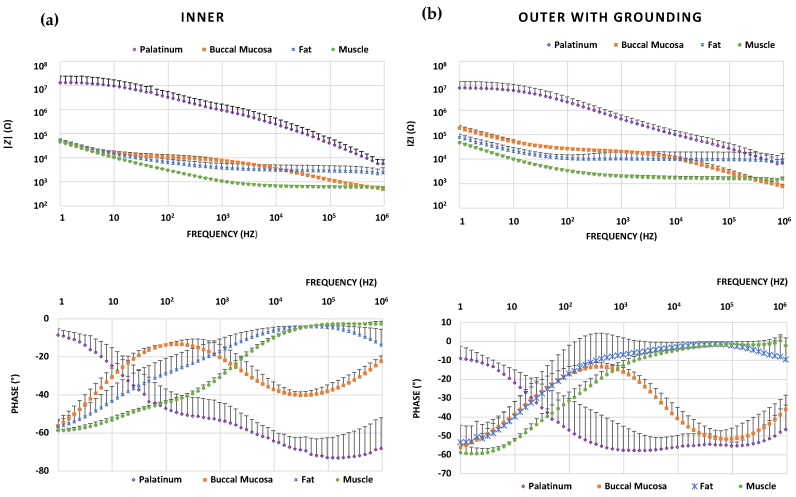
Bode plots of mean BIS spectra for different ex vivo pork oral tissue samples using (**a**) the inner configuration and (**b**) the outer with grounding configuration. Error bars represent the standard deviation of the mean value in one direction.

Figure 6 shows Bode plots of the mean BIS spectra for each tissue type measured by using both the inner and outer with grounding configurations. In all of the tissue types, the general trend was that the impedance magnitude decreased with increasing frequency. All tissues showed characteristic spectra, which differed significantly from other tissue types. Muscle tissue possessed the lowest and palatinum the highest impedance magnitude values. 

At higher frequencies, muscle and fat had the phase values closest to zero among all of the tested tissues. Both the inner and outer with grounding configurations produced similar, tissue-specific spectral shapes. The most consisting results were obtained on muscle and buccal mucosa tissue (all spectra were very near each other), whereas in the case of palatinum and fat there was more diversity between the measurement locations (Table 1 and Table 2).

There were statistically significant differences among tissue types (*p* = 0.001–0.014, Kruskal-Wallis test). The pairwise tissue comparisons (Dunn’s post hoc test) showed that the magnitude and phase differed most frequently among the pairs of palatinum-muscle and palatinum-fat, but most seldomly within the fat-muscle pair (Table 3). 

**Figure 7:**
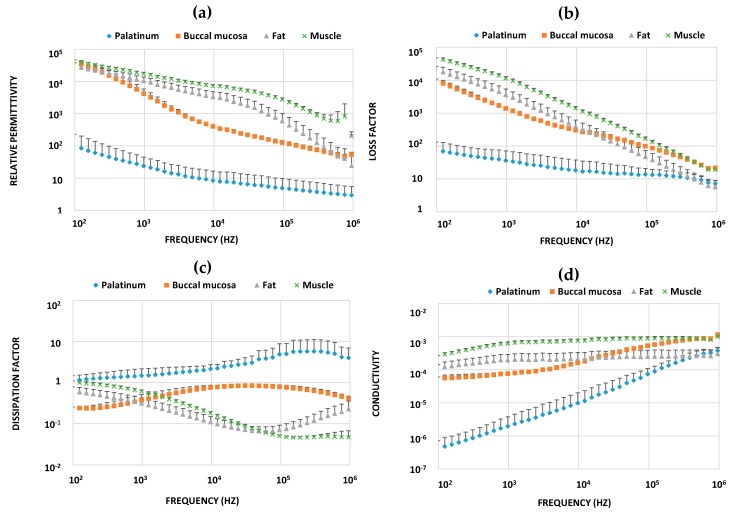
Relative permittivity (**a**), loss factor (**b**), dissipation factor (**c**), and conductivity (**d**) determined for different ex vivo pork oral tissue samples on the basis of BIS measurements using the inner configuration. Data represent mean values and error bars indicate the standard deviation of the mean in one direction.

To further explore the electrical characteristics of different ex vivo pork oral tissue samples, relative permittivity, loss factor, dissipation factor, and conductivity were determined for both the inner configuration (Figure 7) and outer with grounding configuration (Figure S6). These parameters and their frequency-dependencies varied extensively among different tissue types.

**Figure 8:**
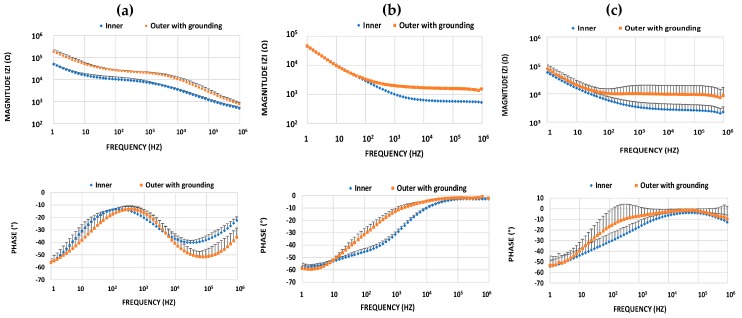
Bode plot (magnitude, phase) comparisons between the inner and outer with grounding configurations separately for (**a**) buccal mucosa, (**b**) muscle, and (**c**) fat. Data represent mean values and error bars indicate the standard deviation of the mean in one direction.

Comparisons between the inner and outer with grounding configurations are given separately for buccal mucosa, muscle, and fat (Figure 8). Regarding magnitude data, the inner configuration resulted in lower impedance magnitudes in all cases. On the other hand, phase analysis showed that fat and muscle behaved in a similar manner; phase tended to reach the zero level at frequencies higher than tens of kHz, and this tendency was stronger when using the outer with grounding configuration. However, the phase data is totally different in the case of buccal mucosa, for which much lower phase values are seen in the kilohertz region. 

## 4. Discussion

The main goal of the present study was to design, test, and optimize a new concentric ring probe for BIS measurements of soft oral tissues. The feasibility testing was conducted with ex vivo pork oral tissue samples. Overall, the obtained results show that multi-frequency BIS with the new concentric probe is a viable option for characterizing and differentiating different tissue types.

Around 1 MHz, there is an abrupt drop in the impedance modulus and a rise in the phase. This might be due to the electrode probe structure and its disturbed operation above 1 MHz due to increased parasitic capacitance [21]. This was verified by examining spectra from an open circuit (air) measurement (data not shown). Therefore, we limited the visualization of spectral images at frequencies below 1 MHz.

Our probe enables BIS measurements to be taken with two different inter-electrode distances. The distance between the electrodes influences the probing depth of the penetration currents inside the tissue samples. Regarding the technical quality and reproducibility of the measurements, we did not find any clear differences between the configurations. Recently, Meaney et al. [22] demonstrated that there is a linear relationship between the penetration depth and the diameter of concentric ring probe. According to their simulation model, the penetration depth in our probe measurements can be estimated to be about 0.63 mm and 1.17 mm, respectively, for inner and outer configuration. Depth information is crucial when studying layered tissues such as the oral mucosa, and this information can be determined using differently spaced concentric ring electrodes.

There are at least two relevant issues affecting the measurement results: appropriate probe material/tissue interface contact and squeezing out the free water/liquid from surface layer. Four-terminal measurement is a commonly used technique in bioimpedance measurements. Separate electrodes for current injection and voltage sensing eliminate the electrode-tissue interface contact impedance from the measurements. This is especially important when measuring impedances at a low frequency region. In the present BIS application, we selected a two-terminal strategy due to its simplicity and on the basis of our hypothesis that higher frequencies (where the importance of contact impedance is lower) would be more useful and possess better distinguishing capability. We noticed that it is important to reach an appropriate contact (pressure) between the measuring probe and tissue sample. In various previous studies, it has been found that the impedance on various tissues increases with the applied load on the measurement probe and can be even seven times higher than that achieved with a lower loading weight [23,24]. For our both non-biological samples, i.e., white tissue paper and yellow towel, the measurement setup without any additional loading weight produced a significantly lower reproducibility than that with 400 g loading weight in all three separate configurations. On the other hand, applying a 100 g loading weight produced measurements with a lower reproducibility than that using a 200 g loading weight. We also found that the relative standard deviation for both samples was lower when using heavier weights (200 g or 400 g) in comparison to lighter weights (100 g or no weight) in all configurations (Figures S2 and S3). 

Phantom materials and biological tissues behave somewhat differently, as we observed. Repeated measurement with biological materials showed that a 400 g loading weight squeezed fluid out from the samples in all configurations; thus, a 200 g loading weight corresponding the pressure around 0.03 MPa showed the most consistent results. A possible explanation for this could be the viscoelasticity of soft tissues, which leads to time-dependent behavior, especially when using heavier loads. Increasing loading increases the compression of the samples. In any biological ex vivo sample, intercellular fluid is compressed with increasing loading weight, which results in an increase in both extracellular resistance and membrane capacitance while reducing the intracellular resistance [25]. On the other hand, it has been reported by researchers that there is a relationship between the probe size and the applied loading, such that a wider electrode is independent of the applied loading effect whereas for a smaller probe it is necessary to apply a uniform loading [23]. Our probe is about 9 mm in diameter, and thus we standardized our measurement protocol to avoid artifacts and maximize the reproducibility of the obtained results [13] by systematically choosing the most consistent loading weight (200 g) for all of the biological samples. On the other hand, because the low frequencies seemed to also be interesting, 4-terminal probe might be well useful, and should be investigated in the future. In this way, we can also reduce the importance of fixed pressure.

Many previous studies have attempted to characterize different biological tissues using needle-type electrodes [15,26,27,28]. However, we used a non-invasive concentric ring-type surface electrode as a novel approach for measuring oral tissues. Previously, such ring electrodes have been applied, e.g., for the detection of emboli in vessels [29], characterization of food [30], and recording of brain signals [31]. The Bode plots of the mean BIS spectra (Figure 6) for ex vivo pork oral tissues show evidence of their significantly different electrical characteristics. By analyzing the impedance magnitude and phase in a certain frequency range, it was possible to differentiate each tissue with statistical significance [15]. In addition to tissue differentiation, BIS is also useful to observe significant changes in the tissue structure when analyzing more precisely the area of interest. This kind of information is useful in various diagnostics applications or during endoscopic operations. The most consistent results were obtained for muscle and buccal mucosa tissue (Table 1 and Table 2), for which RSDs of impedance magnitude varied between 5% and 32% depending on the frequency used, whereas in the case of fat and palatinum, there was more diversity between the measurement locations. Conducted statistical tests (Kruskal-Wallis test) confirmed that there were significant differences among the studied tissue types. Through Dunn’s pairwise tissue comparison (Table 3), we observed that in a certain discrete frequency range it is always possible to discriminate the tissues. In our experiments, magnitude and phase differed most frequently among the pairs of palatinum-fat and palatinum-muscle and most seldomly within the buccal mucosa-fat pair.

There are two frequency-dependent electrical conduction components of biological tissues, i.e., extra- and intracellular spaces, which are separated by insulating membranes [14]. By using the Cartesian form of impedance, it is possible to establish the frequency dependence relationship between impedance Z, conductivity $\sigma^{\prime }$, and relative permittivity $\epsilon_{r}^{\prime }$, which can provide an explanation for the magnitude variability of different tissue types. This relationship is presented in Equation (5):

(5) $$\;Z=R+jX=\frac{1}{\sigma^{\prime }+j\omega \epsilon_{0}\epsilon_{r}^{\prime }}\;$$

where ω is the angular frequency and $\epsilon_{0}$ is the permittivity of free space. 

To further analyze our BIS data on different ex vivo pork oral tissue samples, we calculated conductivity, relative permittivity, loss factor, and dissipation factor (Figure 7 and Figure S4). Relative permittivity and conductivity values varied highly between different tissue types, in concordance with previous reports (e.g., Ref. [14]). Muscle tissues contain more water (around 76%) whereas fat tissues contain less (around 10%), and relative permittivity of water is about 80 [32,33]. Thus, muscle tissues possess higher relative permittivity than fat tissues [34]. Electric pulse causes transient dielectric breakdown and conductivity is increased, which implies a reduction of the impedance magnitude due to the increased permeability of the tissue membrane [14]. The measured impedance magnitude values (Figure 6) were lower for muscle than those for fat, which is consistent with previously published studies [26,35]. This is because in the low frequency regime, the accumulation of lipid inside the cell expands the cell size, which reduces the path for an electrical current due to the smaller extracellular space. In contrast, at high frequencies, the current passes through the intracellular matrix and fat tissue has a higher impedance than the other intracellular substances [28].

Through both in vivo and ex vivo studies, some differences in electrical impedance between normal and cancerous tissues have been found [28]. In some liver diseases such as liver cancer, fatty liver tissues usually resemble a cancerous event and impedance is higher in those tissues [28]. There is some incoherence in the electrical properties (relative permittivity, conductivity, etc.) of cancerous tissues, since some reports have noted that parameters are affected by less than 10%, whereas others have said that the increasing factor is 1.5–2 times compared to normal healthy tissues [36]. Different researchers and probes measure different volume of tissues so large probes measure easily the surrounding health tissues. Since our concentric ring probe seemed to be highly sensitive to differentiating tissue types that possess varying electrical properties, it might also be feasible to characterize cancerous changes in the oral mucosa.

However, we also acknowledge some limitations of our work, e.g., the low number of samples, which were extracted from two animals. Due to the low statistical power, significant differences between the tissue types were not always reached, although the differences seemed to be evident. Therefore, further studies are warranted to clarify the potential of our measurement approach to distinguish different ex vivo and in vivo tissues, also considering tissues with specific pathological (cancerous) tissue alterations. It is evident that demographic and clinical factors such as gender, smoking [37] and salivary flow [13] probably affect bioimpedance results, and they need to be clarified in further studies. When considering in vivo measurements in the oral cavity, more standardized measurements are needed [13] and a new sensor design is required. We acknowledge that there are still a lot of challenges to implement this method in real clinical settings. However, the optimum force can be realized with pressure sensors integrated in a handheld device, such as in the present electric toothbrushes (e.g., Oral-B.). This study was performed by using two terminal measurements with smooth electrode surfaces. In future, we will also test other measurement options, such as four-terminal measurement as well as micro- and nanostructured electrode surfaces [38,39]. These new approaches can also reduce the importance of fixed pressure. One possibility for a suitable sensor design could be a single-use thin-film or screen-printed sensor.

## 5. Conclusions

The functionality of the new concentric ring probe seemed to be adequate for assessing the electrical properties of ex vivo tissue samples. All studied tissues showed their own unique impedance spectra accompanied by significant differences in impedance magnitude and phase. The loading weight and time period under press must to be fixed to allow meaningful quantitative comparison. Further studies are needed not only to clarify the probe’s potential to distinguish different ex vivo and in vivo tissues, but also to consider tissues with specific pathological tissue alterations. Prior to implementing this method for in vivo testing, the new intraoral sensor must be designed and equipped with appropriate technical solutions such as four-terminal measurement principle to reduce the importance of fixed loading pressure. 

## Figures and Tables

**Table 1:** Impedance magnitude and phase values (mean ± stdv) for each tissue type at seven discrete frequencies using the inner configuration. The *p*-value was calculated using the Kruskal-Wallis test.

Frequency	Parameter	Palatinum	Buccal Mucosa	Fat	Muscle	*p*-Value
1 Hz	Magnitude (kΩ)	12,738.3 ± 11,332.5	50.7 ± 2.4	56.1 ± 6.0	44.1 ± 2.3	<0.001
	Phase (°)	−8.6 ± 3.3	−56.1 ± 3.4	−54.3 ± 5.5	−57.9 ± 1.5	0.004
10 Hz	Magnitude (kΩ)	9312.9 ± 7740.8	15.9 ± 2.98	15.8 ± 4.3	10.3 ± 0.6	<0.001
	Phase (°)	−24.6 ± 9.4	−30.6 ± 4.8	−44.0 ± 6.3	−53.2 ± 1.3	<0.001
100 Hz	Magnitude (kΩ)	3187.4 ± 2222.2	10.5 ± 2.9	6.2 ± 2.2	3.0 ± 0.3	<0.001
	Phase (°)	−47.2 ± 10.3	−13.7 ± 0.6	−31.2 ± 7.5	−45.4 ± 2.5	0.002
1 kHz	Magnitude (kΩ)	923.1 ± 731.8	7.6 ± 1.2	3.5 ± 1.7	1.0 ± 0.1	<0.001
	Phase (°)	−53.4 ± 9.5	−20.3 ± 6.8	−17.3 ± 5.8	−31.4 ± 2.1	<0.001
10 kHz	Magnitude (kΩ)	244.4 ± 1925.2	3.5 ± 0.3	2.8 ± 1.6	0.6 ± 0.1	<0.001
	Phase (°)	−64.2 ± 5.4	−37.0 ± 3.9	−6.3 ± 2.2	−10.2 ± 0.9	<0.001
100 kHz	Magnitude (kΩ)	40.1 ± 26.1	1.2 ± 0.2	2.6 ± 1.5	0.6 ± 0.09	<0.001
	Phase (°)	−72.9 ± 10.4	−38.2 ± 3.4	−4.1 ± 1.2	−2.9 ± 0.1	0.001
1 MHz	Magnitude (kΩ)	5.6 ± 2.6	0.5 ± 0.1	2.3 ± 1.2	0.5 ± 0.1	0.001
	Phase (°)	−68.1 ± 16.2	−22.4 ± 2.8	−12.6 ± 6.8	−2.7 ± 1.2	<0.001

**Table 2:** Impedance magnitude and phase values (mean ± stdv) for each tissue type at seven discrete frequencies using the outer with grounding configuration. The *p*-value was calculated using the Kruskal-Wallis test.

Frequency	Parameter	Palatinum	Buccal Mucosa	Fat	Muscle	*p*-Value
1 Hz	Magnitude (kΩ)	8028.1 ± 6948.1	186.2 ± 47.8	76.2 ± 26.6	46.4 ± 5.1	<0.001
	Phase (°)	−9.1 ± 6.6	−9.1 ± 1.1	−53.3 ± 9	−58.9 ± 4.2	0.005
10 Hz	Magnitude (kΩ)	6298.5 ± 5054.1	51.6 ± 10.1	20.6 ± 9.1	9.9 ± 1.2	<0.001
	Phase (°)	−22.0 ± 6.9	−21.9 ± 6.7	−39.0 ± 8.1	−52.4 ± 1.1	0.001
100 Hz	Magnitude(kΩ)	2137.0 ± 1319.5	26.7 ± 2.1	10.5 ± 4.5	3.4 ± 0.4	<0.001
	Phase (°)	−47.0 ± 16.9	−46.9 ± 4.0	−17.1 ± 15.0	−31.5 ± 4.7	0.014
1 kHz	Magnitude (kΩ)	436.7 ± 202.1	20.5 ± 2.1	10.4 ± 9.5	2.0 ± 0.3	<0.001
	Phase (°)	−57.6 ± 12.7	−57.6 ± 2.4	−7.0 ± 8.9	−12.8 ± 2.7	0.002
10 kHz	Magnitude (kΩ)	100.4 ± 48.9	10.8 ± 2.5	9.6 ± 10.1	1.7 ± 0.3	<0.001
	Phase (°)	−55.9 ± 6.2	−55.9 ± 5.9	−2.8 ± 2.4	−4.7 ± 0.8	0.001
100 kHz	Magnitude (kΩ)	27.5 ± 17.5	2.9 ± 0.9	9.4 ± 10.0	1.6 ± 0.3	0.001
	Phase (°)	−55.3 ± 2.2	−55.3 ± 5.7	−2.2 ± 2.1	−1.7 ± 0.2	0.001
1 MHz	Magnitude (kΩ)	6.9 ± 3.4	0.8 ± 0.1	8.6 ± 8.2	1.5 ± 0.3	0.001
	Phase (°)	−46.7 ± 13	−46.7 ± 7.5	−9.5 ± 11.5	−2.2 ± 0.3	0.002

**Table 3:** Pairwise comparison of pork oral tissue samples. Data are based on impedance magnitude or phase values measured using the inner configuration or outer with grounding configuration for each tissue type. Pairwise difference is represented as a *p*-value, calculated using Dunn’s post hoc test. Significantly different pairs are bolded.

**Inner Configuration: Impedance Magnitude**							
**Tissue Comparison**	**1 MHz**	**100 kHz**	**10 kHz**	**1 kHz**	**100 Hz**	**10 Hz**	**1 Hz**
Palatinum-buccal mucosa	**0.003**	**0.032**	0.179	0.327	0.260	0.137	**0.046**
Palatinum-fat	0.286	0.091	**0.029**	**0.016**	**0.021**	**0.037**	0.075
Palatinum-muscle	**<0.001**	**<0.001**	**<0.001**	**<0.001**	**<0.001**	**<0.001**	**<0.001**
Buccal mucosa-fat	**0.032**	0.446	0.663	0.327	0.446	0.828	0.586
Buccal mucosa-muscle	0.790	0.206	**0.037**	**0.014**	**0.021**	0.053	0.158
Fat-muscle	**0.017**	**0.011**	**0.042**	0.072	0.059	**0.034**	**0.014**
**Outer with Grounding Configuration: Impedance Magnitude**							
**Tissue Comparison**	**1 MHz**	**100 kHz**	**10 kHz**	**1 kHz**	**100 Hz**	**10 Hz**	**1 Hz**
Palatinum-buccal mucosa	**0.001**	0.053	0.210	0.210	0.305	0.305	0.305
Palatinum-fat	0.845	0.238	**0.035**	**0.035**	**0.024**	**0.024**	**0.024**
Palatinum-muscle	**0.007**	**<0.001**	**<0.001**	**<0.001**	**<0.001**	**<0.001**	**<0.001**
Buccal mucosa-fat	**0.003**	0.369	0.596	0.596	0.377	0.377	0.377
Buccal mucosa-muscle	0.243	0.225	**0.036**	**0.036**	**0.020**	**0.020**	**0.020**
Fat-muscle	**0.018**	**0.011**	0.069	0.069	0.099	0.099	0.099
**Inner Configuration: Phase**							
**Tissue Comparison**	**1 MHz**	**100 kHz**	**10 kHz**	**1 kHz**	**100 Hz**	**10 Hz**	**1 Hz**
Palatinum-buccal mucosa	0.231	0.327	0.327	**0.004**	**0.001**	0.663	**0.039**
Palatinum-fat	**0.023**	**0.007**	**<0.001**	**<0.001**	**0.008**	**0.029**	**0.009**
Palatinum-muscle	**<0.001**	**<0.001**	**0.008**	0.066	0.449	**<0.001**	**<0.001**
Buccal mucosa-fat	0.514	0.217	**0.014**	0.690	0.260	0.179	0.942
Buccal mucosa-muscle	**0.026**	**0.026**	0.251	0.145	**0.006**	**0.005**	0.483
Fat-muscle	0.053	0.230	0.087	**0.021**	**0.044**	0.072	0.437
**Outer with Grounding Configuration: Phase**							
**Tissue Comparison**	**1 MHz**	**100 kHz**	**10 kHz**	**1 kHz**	**100 Hz**	**10 Hz**	**1 Hz**
Palatinum-buccal mucosa	0.543	0.649	0.210	0.176	**0.008**	0.184	**0.044**
Palatinum-fat	**0.021**	**0.010**	**0.000**	**0.003**	**0.008**	0.072	**0.011**
Palatinum-muscle	**<0.001**	**<0.001**	**0.002**	**0.002**	0.258	**<0.001**	**0.001**
Buccal mucosa-fat	0.185	0.091	0.146	0.077	0.702	0.837	0.883
Buccal mucosa-muscle	**0.020**	0.142	0.159	0.243	0.070	0.052	0.470
Fat-muscle	0.271	0.422	0.828	0.409	0.097	**0.042**	0.504

## Supplementary Materials

The following are available online at http://www.mdpi.com/1424-8220/18/10/3378/s1, Figure S1: Examples of pork oral tissue samples: (a) palatinum, (b) buccal mucosa, (c) fat, and (d) muscle with marked measurement locations. Figure S2: Relative standard deviation (RSD) of impedance magnitude and phase in repeated bioimpedance spectroscopy (BIS) measurements for white tissue paper using (a) the inner configuration, (b) the outer configuration, and (c) the outer with grounding configuration. Figure S3: Relative standard deviation (RSD) of impedance magnitude and phase in repeated BIS measurements for yellow towel using (a) the inner configuration, (b) the outer configuration, and (c) the outer with grounding configuration. Figure S4: Bode plots for repeated measurements on the same (a) cucumber and (b) tongue sample using inner configuration. Figure S5: Bode plots for repeated measurements on the same (a) cucumber and (b) tongue sample using outer with grounding configuration. Figure S6: Relative permittivity (a), loss factor (b), dissipation factor (c), and conductivity (d) determined for different ex vivo pork oral tissue samples on the basis of BIS measurements using the outer with grounding configuration. Data represent mean values and error bars indicate the standard deviation of the mean in one direction.
