# Supplementary material for: Concentric Ring Probe for Bioimpedance Spectroscopic Measurements: Design and Ex Vivo Feasibility Testing on Pork Oral Tissues
Source: Sensors (Basel). 2018 Oct 10;18(10):3378. doi: 10.3390/s18103378 (PMC6210762; doi:10.3390/s18103378)
Supplement: Supplementary file 1 [file sensors-18-03378-s001.pdf]

Supplementary material

Concentric Ring Probe for Bioimpedance Spectroscopic Measurements: Design and Ex Vivo Feasibility Testing on Pork Oral Tissues

Shekh Emran [1], Reijo Lappalainen [1], Arja M. Kullaa [2,3,4] and Sami Myllymaa [1,*]

1 SIB Labs, Department of Applied Physics, University of Eastern Finland, P.O. Box 1627, FI-70211 Kuopio, Finland; shekh.emran@uef.fi (S.E.); reijo.lappalainen@uef.fi (R.L.)
2 Institute of Dentistry, University of Eastern Finland, P.O. Box 1627, FI-70211 Kuopio, Finland; arja.kullaa@uef.fi
3 Research Unit of Oral Health Sciences, University of Oulu, P.O. Box 8000, FI-90014 Oulu, Finland
4 Educational Dental Clinic, Kuopio University Hospital, P.O. Box 100, FI-70029 Kuopio, Finland
* Correspondence: sami.myllymaa@uef.fi; Tel.: +358-40-557-2499

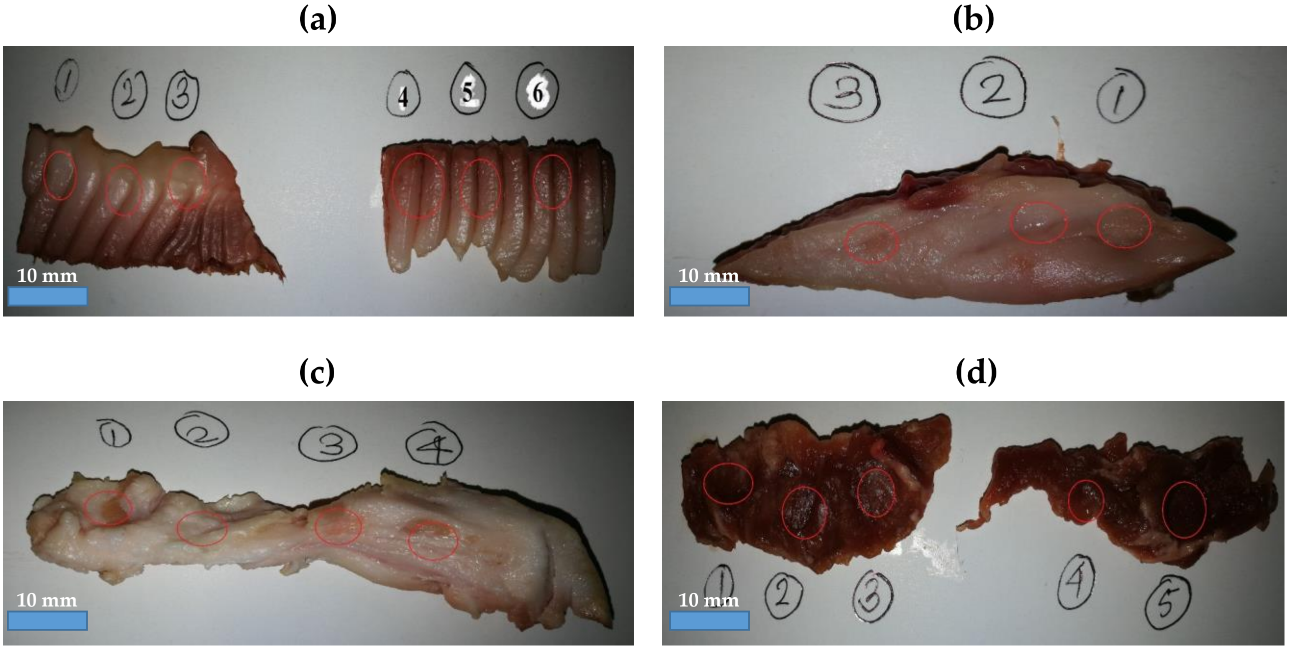

Figure S1. Examples of pork oral tissue samples: (a) palatinum, (b) buccal mucosa, (c) fat and (d) muscle with marked measurement locations.

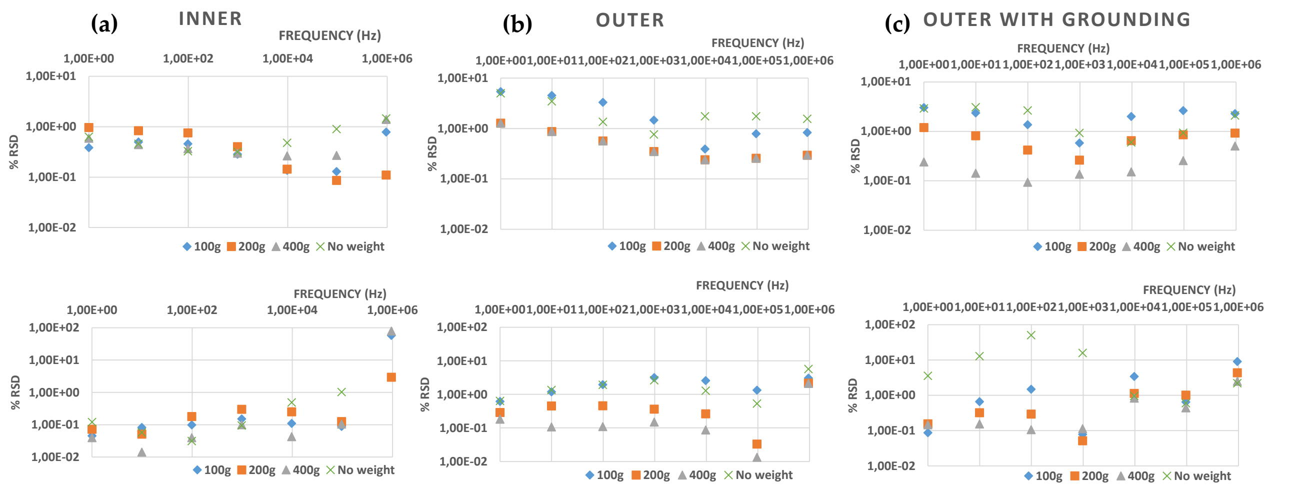

Figure S2. Relative standard deviation (RSD) of impedance magnitude and phase in repeated bioimpedance spectroscopy (BIS) measurements for white tissue paper using (**a**) the inner configuration, (**b**) the outer configuration, and (**c**) the outer with grounding configuration.

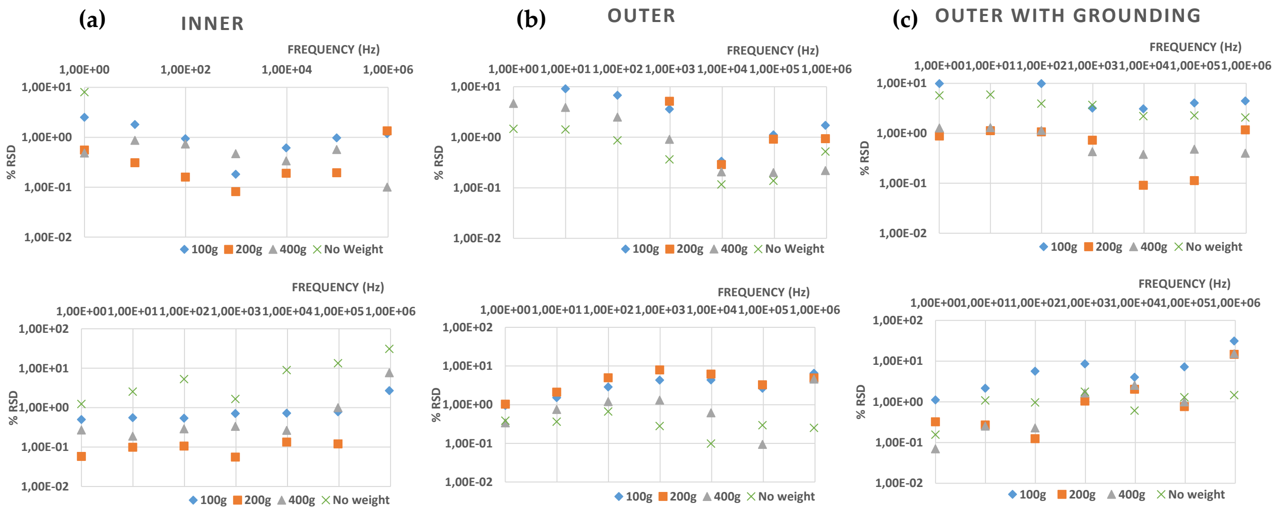

Figure S3. Relative standard deviation (RSD) of impedance magnitude and phase in repeated BIS measurements for yellow towel using (**a**) the inner configuration, (**b**) the outer configuration, and (**c**) the outer with grounding configuration.

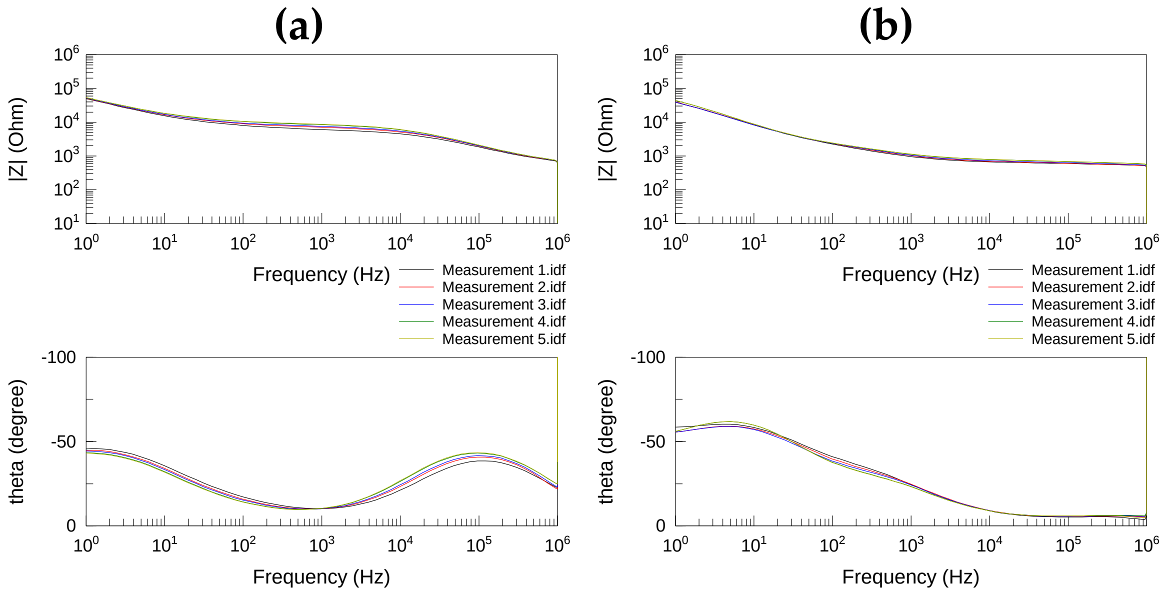

Figure S4. Bode plots for repeated measurements on the same (a) cucumber and (b) tongue sample using inner configuration.

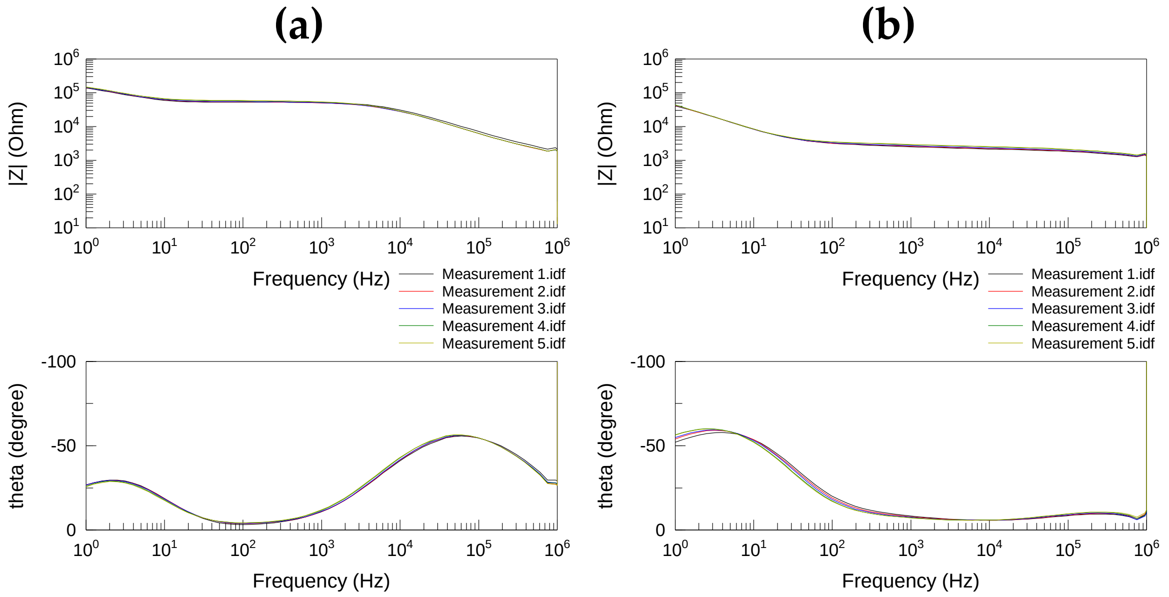

Figure S5. Bode plots for repeated measurements on the same (a) cucumber and (b) tongue sample using outer with grounding configuration.

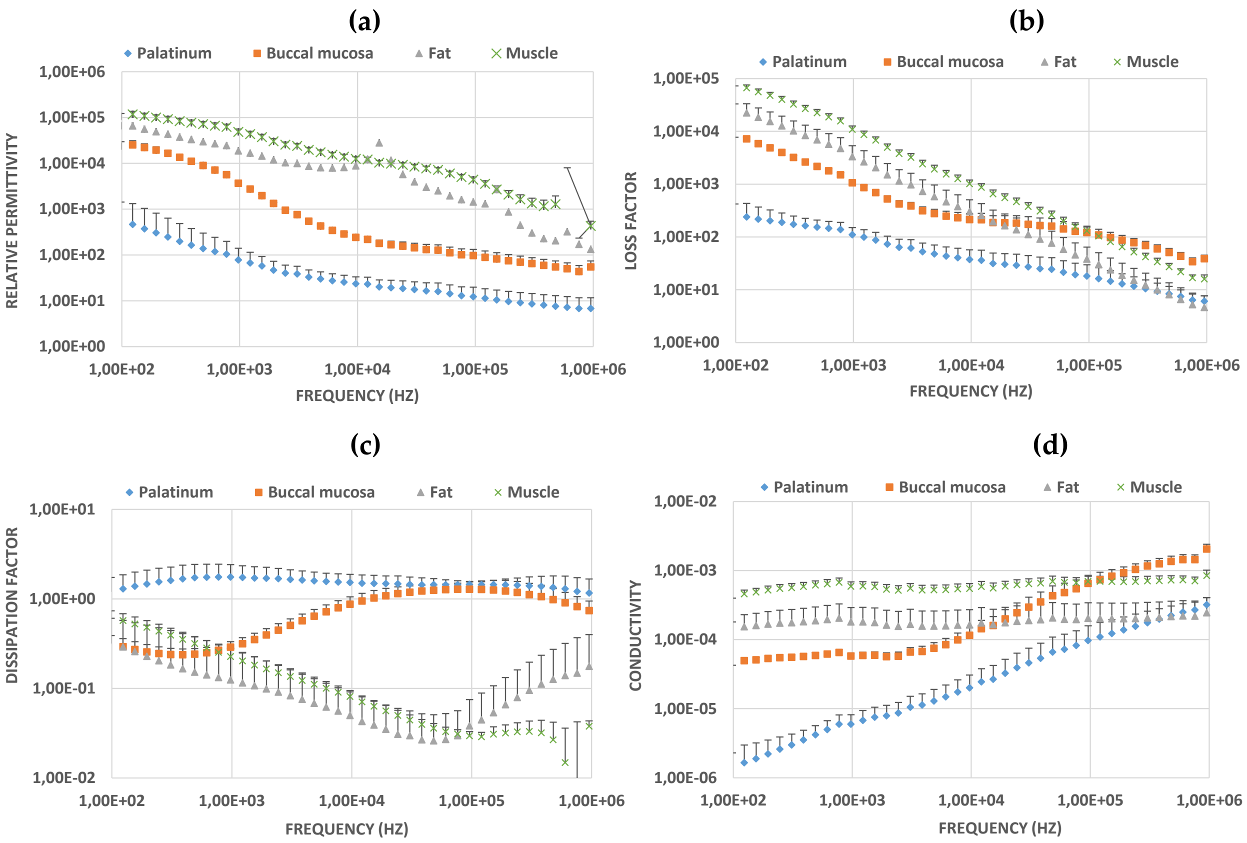

Figure S6. Relative permittivity (a), loss factor (b), dissipation factor (c), and conductivity (d) determined for different ex vivo pork oral tissue samples on the basis of BIS measurements using the outer with grounding configuration. Data represent mean values and error bars indicate the standard deviation of the mean in one direction.
